# Excess DHA Induces Liver Injury *via* Lipid Peroxidation and Gut Microbiota-Derived Lipopolysaccharide in Zebrafish

**DOI:** 10.3389/fnut.2022.870343

**Published:** 2022-04-28

**Authors:** Qianwen Ding, Qiang Hao, Qingshuang Zhang, Yalin Yang, Rolf Erik Olsen, Einar Ringø, Chao Ran, Zhen Zhang, Zhigang Zhou

**Affiliations:** ^1^China-Norway Joint Lab on Fish Gastrointestinal Microbiota, Institute of Feed Research, Chinese Academy of Agricultural Sciences, Beijing, China; ^2^Norway-China Joint Lab on Fish Gastrointestinal Microbiota, Institute of Biology, Norwegian University of Science and Technology, Trondheim, Norway; ^3^Key Laboratory for Feed Biotechnology of the Ministry of Agriculture and Rural Affairs, Institute of Feed Research, Chinese Academy of Agricultural Sciences, Beijing, China; ^4^Faculty of Bioscience, Fisheries and Economics, Norwegian College of Fishery Science, UiT the Arctic University of Norway, Tromsø, Norway

**Keywords:** DHA, liver, apoptosis, gut microbiota, lipopolysaccharide

## Abstract

Being highly unsaturated, *n*-3 long-chain polyunsaturated fatty acids (LC-PUFAs) are prone to lipid peroxidation. In this study, zebrafish were fed with low-fat diet (LFD), high-fat diet (HFD), or 2% DHA-supplemented HFD (HFDHA2.0). To study the possible negative effects of the high level of dietary DHA, growth rates, blood chemistry, liver histology, hepatic oxidative stress, apoptosis, and inflammatory processes were assessed. The cell studies were used to quantify the effects of DHA and antioxidant on cellular lipid peroxidation and viability. The possible interaction between gut microbiota and zebrafish host was evaluated *in vitro*. HFDHA2.0 had no effect on hepatic lipid level but induced liver injury, oxidative stress, and hepatocellular apoptosis, including intrinsic and death receptor-induced apoptosis. Besides, the inclusion of 2% DHA in HFD increased the abundance of Proteobacteria in gut microbiota and serum endotoxin level. In the zebrafish liver cell model, DHA activated intrinsic apoptosis while the antioxidant 4-hydroxy-Tempo (tempo) inhibited the pro-apoptotic negative effects of DHA. The apoptosis induced by lipopolysaccharide (LPS) was unaffected by the addition of tempo. In conclusion, the excess DHA supplementation generates hepatocellular apoptosis-related injury to the liver. The processes might propagate along at least two routes, involving lipid peroxidation and gut microbiota-generated LPS.

## Introduction

Farmed fish fed with marine oils rich in docosahexaenoic acid (DHA, C22:6) and eicosapentaenoic acid (EPA, C20:5) generate high-quality products with long-chain polyunsaturated fatty acids (LC-PUFAs) (1). To ensure the nutritional quality of farmed fish based on *n*-3 LC-PUFAs contents, those levels supplemented into fish diets are in excess of those required for optimal growth and health of fish (2). The negative effects of excess dietary *n*-3 LC-PUFAs have been revealed in few fish species, like Atlantic bluefin tuna (*Thunnus thynnus*) and grass carp (*Ctenopharyngodon idella*), which are characterized by higher mortality, poor growth performance, and hepatic pathology (3, 4). Although *n*-3 LC-PUFAs have unique physical properties that appear to influence cell membrane structure and cell signaling transduction (5), it has been suggested that their high number of methylene interrupted double bonds make them susceptible to free radical attack (6, 7). Mammalian studies have suggested that increasing intake of DHA enhances susceptibility of the liver to lipid peroxidation concomitant with higher deposition of DHA (8–11). Moreover, DHA might be a pro-apoptotic factor that can induce lipid peroxidation (12–14). Likewise, high levels of dietary LC-PUFAs increase the deposition of DHA in juvenile Arctic char (*Salvelinus alpinus*) in both the liver and muscle along with indices of oxidative stress (15). Free radical injury in the muscle has been reported in fin sea bass (*Dicentrarchus labrax*) larvae fed high dietary DHA (16). However, the action mode underlying the damage caused by DHA in fish is poorly understood.

Mammalian studies have suggested that appropriate levels of DHA could improve the gut microbiota disorder caused by high-fat diet (HFD) or chronic stress (17–20). The health-promoting effects of DHA, such as alleviating hyperglycemia, insulin resistance, and lipid metabolism abnormalities, are partly mediated by the modulation of gut microbiome and bacterial metabolites (21–23). The previous works in zebrafish also observed a similar phenomenon (24, 25). However, in the case of high dietary DHA, the available information is scarce both in mammals and fish. Nevertheless, few fish studies have suggested that high levels of *n*-3 LC-PUFAs in high-lipid diet induce a less healthy gut microbiota community characterized by the reduction of lactic acid bacteria and higher abundance of *Mycoplasma, Burkholderiaceae, Bacteroidales*, and *Ralstonia* in Atlantic Salmon (*Salmo salar)* and hybrid grouper (female *Epinephelus fuscoguttatus* × male *Epinephelus lanceolatu*) (26, 27). In contrast, a study in gilthead sea bream (*Sparus aurata*) suggested that *n*-3 LC-PUFAs could promote the growth of probiotics by reducing the diversity of microbe (28). To clarify the effects of high dietary DHA on gut microbiota, and the links between excess DHA, gut microbiota, and fish health, further studies are needed.

Zebrafish (*Danio rerio*) are evolutionally closer to the most important cultured fish and have been increasingly used for nutritional evaluation of aquatic feeds (29, 30). Moreover, the tissue deposition of DHA and oxidative stress induced by dietary DHA have been observed in zebrafish (31). A previous study has suggested that short-term (2 weeks) and medium-term (4 weeks) intakes of low level of DHA (0.5% of diet) are beneficial for reducing hepatic lipid accumulation induced by HFD, however, as the inclusion of DHA increased to 2.0% of diet, this lipid-lowering effect was abolished (24). Thus, in this study, the effects of high dietary DHA (2.0% of diet) on liver health as well as hepatic oxidative stress were studied. Moreover, the relationship between liver injury and lipid peroxidation was validated by *in vitro* supplementation of the antioxidant 4-hydroxy-Tempo (tempo). Finally, a possible interaction between gut microbiota and zebrafish host was evaluated using *in vitro* assays. This study is beneficial for revealing the possible risks of supplementing high levels of DHA to fish.

## Materials and Methods

### Fish Husbandry

Zebrafish of the Tübingen strain were maintained at the zebrafish facility of the Institute of Feed Research, Chinese Academy of Agricultural Sciences (Beijing, China) for 4 weeks as described in a previous study (32). The size of each tank was 25.5 × 18.5 × 18.0 cm. During the feeding period, the water in the rearing system was kept running, the rearing temperature was 25–28°C, the dissolved oxygen was >6.0 mg/L, the pH was 7.0–7.2, the nitrogen content was <0.50 mg/L and the nitrogen content (as NO_2_) was <0.02 mg/L. Zebrafish were maintained at a 14:10 L:D cycle.

### Diets and Feeding Trial

The feed formulation is presented in Table 1. The feeds were prepared as described in previous work (24). Casein, soybean oil, and wheat flour were used as dietary protein, lipid, and carbohydrate sources, respectively. The low-fat diet (LFD) was supplemented with soybean oil with 60 g/kg and the HFD was supplemented with soybean oil with 160 g/kg (Table 1). The DHA oil used in the feeding trial has a purity of 90% (Larodan, 10-2206-90-13). The DHA was compensated by decreasing equal level of soybean oil. All dry ingredients were ground through a 60-mesh screen. The diets were prepared by mixing the dry ingredients with the oil and water manually. Then each diet was extruded in a manual extruder with a 2.5-mm aperture. The extruded pellets were freeze-dried and stored at −20°C in plastic bags in small quantities. The feed pellets were ground through a 30-mesh screen prior to feeding.

**Table 1:** Ingredients of experimental diets for one-month-old zebrafish (g/kg).

	**One-month-old**			
	**LFD**	**LFDHA2.0**	**HFD**	**HFDHA2.0**
**Ingredients (g/kg dry diet)**
Casein	400	400	400	400
Geltin	100	100	100	100
Wheat flour	350	350	250	250
DHA^a^	0	20	0	20
Soybean oil	60	40	160	140
Lysine	3.3	3.3	3.3	3.3
Ascorbyl phosphate	1	1	1	1
Vitamin premix^b^	2	2	2	2
Mineral premix^c^	2	2	2	2
Monocalcium phosphate	20	20	20	20
Choline chloride	2	2	2	2
Sodium alginate	20	20	20	20
Microcrystalline cellulose	39.7	39.7	39.7	39.7
Total	1,000	1,000	1,000	1,000
**Proximate composition** (g/kg dry diet)				
Crude protein	458.9	459.2	459.3	463.6
Crude lipid	57.3	57.7	152.3	152.0
Ash	31.1	31.4	31.2	32

a *Larodan.*

b *Vitamin premix (g/kg): thiamine, 0.438; riboflavin, 0.632; pyridoxine·HCl, 0.908; d-pantothenic acid, 1.724; nicotinic acid, 4.583; biotin, 0.211; folic acid, 0.549; vitamin B-12, 0.001; inositol, 21.053; menadione sodium bisulfite, 0.889; retinyl acetate, 0.677; cholecalciferol, 0.116; dl-α-tocopherol-acetate, 12.632.*

c *Mineral premix (g/kg): CoCl_2_·6H_2_O, 0.074; CuSO_4_·5H_2_O, 2.5; FeSO_4_·7H_2_O, 73.2; NaCl, 40.0; MgSO_4_·7H_2_O, 284.0; MnSO_4_· H_2_O, 6.50; KI, 0.68; Na_2_SeO_3_, 0.10; ZnSO_4_·7H_2_O, 131.93; Cellulose, 501.09.*

*LFD, low fat diet; HFD, high-fat diet; HFDHA2.0, 2% DHA-supplemented HFD*.

During the feeding, healthy, uniformly sized 1-month-old zebrafish (1.57 ± 0.011 g/20 fish) were divided into four groups at random and fed with the LFD, 2.0% DHA oil-supplemented LFD (LFDHA2.0), HFD, or 2.0% DHA oil-supplemented HFD (HFDHA2.0) (Table 1). The fatty acid compositions of diets have been described in previous work (24). The feeding amount was at 6% of body weight, twice a day at 9:00 and 16:00. Each group contained three tanks with 20 fish per tank.

### Sample Collection and Analysis

All fish were anesthetized with tricaine methanesulfonate (MS222) before sampling. At the end of the feeding, the fish in each tank were weighed to calculate weight gain (100 × [final body weight — initial body weight]/initial body weight) and feed efficiency (100 × [final body weight – initial body weight]/feed intake). The livers were collected for detection of alanine transaminase (Alt) and aspartate transaminase (Ast) activities. The livers were collected for analysis of histology (H&E and tunnel assay), triacylglycerol (TG) content, enzyme activity, parameters related to oxidative stress, and gene expression. To collect enough samples, the feeding trial was conducted for three times.

### Histological Analysis

A histological analysis of livers was conducted as described in a previous study (24). In brief, the livers of zebrafish were rinsed with sterilized phosphate buffer solution (PBS), fixed in 4% formalin solution, and embedded in paraffin. The liver sections prepared from the paraffin blocks were subjected to H&E and tunnel staining. Zeiss microscope (AXIO Observer A1) was used for obtaining the images.

### TG and Fatty Acids Composition Detection

Hepatic TG was extracted by chloroform:methanol (2:1) as described in a previous study (24). The TG contents were determined by a quantitative enzymatic method as described in a previous study (33) and they were expressed as TG weight per gram protein (mg/g protein). The composition of hepatic fatty acids was detected as described in a previous study (34). In brief, total lipids were extracted from liver freeze-dried powder in chloroform/methanol (2:1, v/v), methylated, and trans-esterified with boron trifluoride in methanol to generate fatty acid methyl esters (FAMEs), which then were separated and quantified by GC (Agilent 6890; Agilent, Savage, MD, USA). Individual methyl esters were identified by comparison to known standards.

### Detection of Serum Alt and Ast

The blood samples of the zebrafish were collected as described in a previous study (34). The Alt and Ast activities were detected as described in a previous study (32). The serum Alt and Ast activities were examined at 510 nm according to the manufacturer's instructions and expressed as enzyme activity units per liter (U/L).

### Detection of Serum Endotoxin

The serum endotoxin levels were determined using the ToxinSensorTM Chromogenic LAL Endotoxin Assay Kit (Genscript, Jiangsu Province, China) according to the manufacturer's instructions. In brief, serum samples were dispensed into endotoxin-free vials and then incubated with LAL and chromogenic substrate consecutively. After incubation, a stopping solution was added to each reaction vial. The absorbance of each sample was read with the SynergyMX Multi-Functional MPP Detector (Biotek, USA) at 545 nm. The serum level of endotoxin in adult zebrafish was expressed as endotoxin units per milliliter (EU/ml).

### Evaluation of Total Antioxidant Capacity

The total antioxidant capacity (T-AOC) was evaluated according to a previous study (35). The ferric ion reducing antioxidant power (FRAP) working solution was freshly prepared by mixing tripyridyltriazine (TPTZ) dilution solution, TPTZ solution, and detection buffer solution at a ratio of 10:1:1 (v/v/v) (35). The FRAP working solution (180 μl) and a sample (5 μl) were mixed and then incubated at 37°C for 5 min (35). Fresh livers were homogenized in ice-cold PBS to release the antioxidants. The homogenate was then centrifugated at 12,000 g for 5 min, and the supernatant was collected for subsequent analysis. The T-AOC was measured by the production of blue Ferric-TPTZ resulting from the reduction of Fe^3+^ TPTZ complex under acidic conditions. The optical density was measured at 593 nm. The T-AOC was defined as the production of FeSO_4_ per gram protein (mmol FeSO_4_/g protein).

### Detection of Malondialdehyde (MDA) and Reactive Oxygen (ROS)

Fresh livers and zebrafish liver (ZFL) cells were homogenized in ice-cold MDA lysis buffer and centrifuged at 13,000 g for 10 min to remove insoluble material. The lipid peroxidation was determined by the reaction of MDA with thiobarbituric acid (TBA) using a lipid peroxidation assay kit according to the manufacturer's instructions (Beyotime Biotechnology, Shanghai, China). In brief, the supernatant was collected and incubated with the TBA solution at 95°C for 60 min to generate MDA–TBA adduct. The optical density of the MDA–TBA adduct was examined at 532 nm. The lipid peroxidation was expressed as MDA content per gram protein (μmol/g protein).

Fresh livers were collected from zebrafish and homogenized for 10 times in the PBS using a glass homogenizer. The homogenate was then centrifugated at 400 g for 10 min. The supernatant was then incubated with a fluorescent probe (Sigma, USA) for 2 h in a 96 well black flat–bottom plate. The fluorescence intensity was measured excitation 490 nm and emission 520 nm. The ROS level was expressed as the fold change compared to the HFD group. The mean fluorescence intensity of ROS signal in ZFL cells was detected by using a fluorescent probe DCFH-DA (Beyotime Biotechnology, Shanghai, China). After the exposure to DHA and tempo, ZFL cells were incubated with DCFH-DA for 30 min and then harvested for fluorescence detection. The analysis was conducted by the Guava easyCyte Flow Cytometer (Merck Millipore, Stafford, VA, USA). The mean fluorescence intensity of the ROS signal was expressed MFI/5,000 cells.

### Detection of Caspase Activity

The activities of caspase-9, caspase-8, caspase-6, and caspase-3 were determined using assay kits (Beyotime Biotechnology, Shanghai, China) as described in a previous study (32). The optical density of the reaction products was examined at 405 nm. The enzyme activity units were expressed as the rate of *p*-nitroaniline (*p*NA) (μmol) released from the substrate per gram protein (μmol pNA released per min/g protein).

### Cell Culture

The ZFL cell line was purchased from American Type Culture Collection (Manassas, VA, USA), and cultured according to established protocols (24, 36). All media were obtained from Corning, Inc. Penicillin–Streptomycin solution and bovine insulin were purchased from Sigma (St. Louis, MO, USA). Murine epidermal growth factor was purchased from Peprotech (Rocky Hill, NJ, USA). Rainbow trout (*Oncorhynchus mykiss*) serum was purchased from Caisson Labs (USA).

### DHA and Lipopolysaccharide (LPS) Solution Preparation

The stock solution of DHA (D2534, Sigma) was prepared by dilution in 5% fatty acid-free BSA and vortexed vigorously. The stock solution of LPS (*Escherichia coli* O55:B5, L2880, Sigma) was prepared in sterile PBS. The prepared DHA (10 mM) and LPS (5 mg/ml) solutions were kept in storage at −20°C.

### Cell Viability Analysis

Cells were first seeded on black 96-well plates (Corning) and incubated for 24 h to sub-confluence. Then the media was removed, and the cells were exposed to fresh media with different concentrations of DHA (0, 50, 100, 200 or 400 μM), tempo (100 μM) or LPS (0, 20, 40, 80, 100, 150, 200 or 250 μg/ml) for 24 h. At the end of the exposure period, the media with DHA was removed, and 100 μl of fresh media with 10% AlarmaBlue cell viability reagent (Invitrogen, USA) was added to each well. After incubation for 1 h, fluorescence was measured with the SynergyMX Multi-Functional MPP Detector (Biotek, USA) at excitation and emission wavelengths of 485 and 595 nm. The ratio of cell viability was calculated using the fluorescence readings of the control and treatments.

### Cell Apoptosis Analysis With Flow Cytometry

The cell apoptosis detection was performed with Annexin V-fluorescein isothiocyanate (FITC) kits (Sigma) as described in a previous study (24). After indicated treatments, cells were collected and incubated with Annexin V-FITC and propidium iodide (PI) in binding buffer in the dark for 10 min at room temperature. The analysis was conducted by the Guava easyCyte Flow Cytometer (Merck Millipore, Stafford, VA, USA). The fluorescence intensity was measured at an excitation wavelength of 488 nm using GRN (525 nm) and RED (690 nm) filters. Data analysis was performed using Flow Guava software.

### Gut Microbiota Analysis

The gut contents were collected as described in a previous study (24). The total bacteria DNA was extracted by using a commercial kit (E.Z.N.A.^®^ soil DNA kit, Omega Bio-tek, Norcross, GA, U.S.) according to instruction. The 16s V3–V4 region was amplified using the primer pairs 338F (5'-ACTCCTACGGGAGGCAGCAG-3') and 806R (5'-GGACTACHVGGGTWTCTAAT-3'). The 16S ribosomal RNA gene sequencing was performed by Majorbio Bio-Pharm Technology Co. Ltd. (Shanghai, China) using Illumina MiSeq PE300 platform (Illumina, San Diego, USA). Then the raw pair-end readings were subjected to a quality–control procedure using the UPARSE-operational taxonomic unit (OTU) algorithm (37). The qualified reads were clustered to generate OTUs at the 97% similarity level using the USEARCH sequence analysis tool (37). A representative sequence of each OTU was assigned to a taxonomic level in the Ribosomal Database Project (RDP) database using the RDP classifier (38).

### Total RNA Extraction, Reverse Transcription, and *q*PCR

Total RNA extraction, reverse transcription, and quantitative polymerase chain reaction (*q*PCR) were conducted according to the previous description (24). Total RNA was isolated using Trizol reagent and then reversed transcribed to cDNA. The *q*PCR was performed using SYBR^®^Green Supermix according to the manufacturer's instructions (TIANGEN, Beijing, China). The results were stored, managed, and analyzed using LightCycler 480 software (Roche, Basel, Switzerland). The *q*PCR primers are listed in Table 2.

**Table 2:** Quantitative PCR primers.

	**Sense (5'−3')**	**Antisense (5'−3')**
*rps11*	Acagaaatgccccttcactg	Gcctcttctcaaaacggttg
*bid*	Tggtgctcctttcctttctt	Aggtcgctggtggactatgt
*bik*	Ggggacgaaatggacaataaa	Ctgcgagaccagtcagaaaca
*bmf1*	Gggtcacgcaacggtatg	Gtccgatgagggtttccac
*bmf2*	Actgctggctctgtcctcaa	Gaacctccacgctaatccct
*noxa*	Aagagcaaaccgctgtagtaga	Catcgcttcccctccatt
*bax2*	Caataagcaacagccaggacc	Gccaccagtgaaggcaaaca
*bok1*	Gccgctcctcagtgtttgc	Agacccgtgttctggtttcg
*mcl1a*	Aactccatcacgccatacc	Tctgctcagccaccctct
*mcl1b*	Taccgtcctcgccttcg	Tgtccacaacccgcctc
*nr13*	Agaactggtgggagatggg	Gttgtcttgcgttgtgga
*nfkb*	Gcaagatgagaacggagacac	Ctaccagcaatcgcaaacaa
*tnfa*	Gtttatcagacaaccgtggca	Ccttcttcgtttggcttcatc
*il-1β*	Tggacttcgcagcacaaaatg	Gttcacttcacgctcttggatg
*il-6*	Cacggaaagatgtctaacgc	Ggatagggaagtgctggatg
*il-10*	Tcacgtcatgaacgagatcc	Cctcttgcatttcaccatatcc

### Data Analysis

The statistical analyses were conducted using GraphPad Prism 5 software (GraphPad Software Inc., San Diego, CA, USA). Results are expressed as the means ± standard errors of the means (SEMs). Comparison between two groups were analyzed using Student's *t*-test. Comparison between multiple groups were analyzed using one-way ANOVA followed by Duncan's test. The statistical significance was set at *p* < 0.05.

## Results

### The Effects of DHA Supplementation on Growth and Hepatic Steatosis

**Figure 1:**
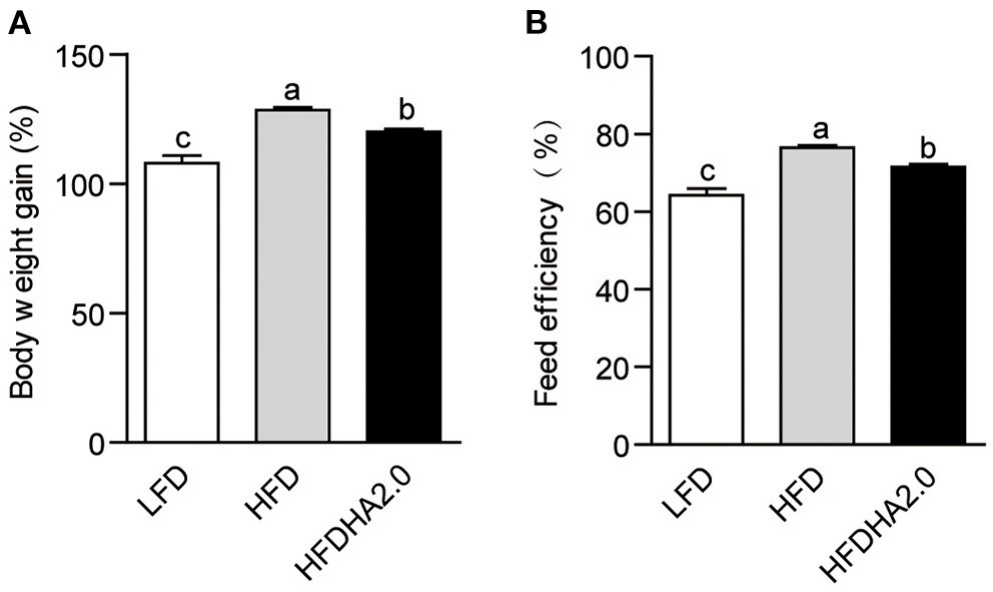
The effects of DHA supplementation on growth and feed efficiency in zebrafish. **(A)** Body weight gain and **(B)** feed efficiency of LFD, HFD, or HFDHA2.0-fed zebrafish. Values are means ± SEMs (*n* = three biological replicates). The mean values without a common letter are significantly different, *p* < 0.05. LFD, low-fat diet; HFD, high-fat diet; HFDHA2.0, 2% DHA-supplemented HFD.

After feeding, body weight gain and feed efficiency were analyzed. Zebrafish fed with HFD had significantly higher body weight gain and feed efficiency as compared with fish fed with LFD (*p* < 0.05; Figures 1A,B). HFDHA2.0 feeding led to significantly lower body weight gain (*p* < 0.05; Figure 1A) and feed efficiency (*p* < 0.05; Figure 1B) than HFD. However, H&E staining (Supplementary Figure 1A) combined with TG quantification (Supplementary Figure 1B) showed similar levels of vacuole-like denaturation and lipid accumulation between HFD and HFDHA2.0 groups, suggesting HFDHA2.0 had no protective effect on fatty liver induced by HFD.

Compared with HFD feeding, HFDHA2.0 significantly decreased saturated fatty acids (SFAs) (26.2 vs. 23.0%) and monounsaturated fatty acids (MUFAs) (26.04 vs. 24.45%) of total hepatic fatty acids (*p* < 0.05; Table 3). The total PUFAs in zebrafish fed with the HFDHA2.0 were significantly increased compared to fish fed with the HFD (52.54 vs. 47.75%, *p* < 0.05; Table 3). However, zebrafish in the LFDHA2.0 group had similar body weight gain and feed efficiency as compared to those in the LFD group (Supplementary Figures 2A,B). These results suggest that a high level of DHA could inhibit growth performance and alter the hepatic fatty acid composition.

**Table 3:** Fatty acid composition in the livers of one-month-old zebrafish fed with HFD or HFDHA2.0 for 4 weeks.

**g/100 g total fatty acid**	**HFD**	**HFDHA2.0**
C14:0	0.66 ± 0.011	0.58 ± 0.024
C15:0	0.21 ± 0.002	0.22 ± 0.020
C16:0	16.89 ± 0.058	15.02 ± 0.038^*^
C17:0	0.42 ± 0.002	0.35 ± 0.001^*^
C18:0	6.80 ± 0.011	5.70 ± 0.003^*^
C20:0	0.20 ± 0.007	0.23 ± 0.019
C21:0	0.57 ± 0.001	0.47 ± 0.026
C22:0	0.21 ± 0.011	0.22 ± 0.001
Total saturates^a^	26.20 ± 0.066	23.00 ± 0.045^*^
C16:1	1.23 ± 0.011	0.98 ± 0.011^*^
C18:1	24.31 ± 0.021	23.03 ± 0.072^*^
C20:1	0.36 ± 0.001	0.32 ± 0.017
Total monoenes^b^	26.04 ± 0.009	24.45 ± 0.043^*^
C18:2	35.95 ± 0.069	40.52 ± 0.129^*^
C20:3	2.11 ± 0.004	0.91 ± 0.014^*^
C20:4	2.09 ± 0.017	0.69 ± 0.016^*^
Total (*n*-6)^c^	40.15 ± 0.091	42.12 ± 0.099^*^
C18:3	3.46 ± 0.034	4.54 ± 0.034^*^
C20:5	0.66 ± 0.001	1.00 ± 0.01^*^
C22:6	3.36 ± 0.001	4.77 ± 0.051^*^
Total (*n*-3)^d^	7.61 ± 0.033	10.42 ± 0.097^*^
Total PUFAs	47.75 ± 0.057	52.54 ± 0.002^*^
(*n*-3):(*n*-6)	0.19 ± 0.001	0.25 ± 0.003^*^

*Values are means ± SEMs; n = 2.*

* *p < 0.05.*

a *includes 6:0, 10:0, 12:0, 14:0, 15:0, 16:0, 17:0, 18:0, 20:0, 21:0, 22:0, 24:0.*

b *includes 14:1, 16:1, 18:1, 20:1, 22:1, 24:1.*

c *includes 18:2, 20:3, 20:4.*

d *includes 18:3, 20:3, 20:5, 22:6.*

*HFD, high-fat diet; HFDHA2.0, 2% DHA-supplemented HFD*.

### The Effects of DHA on Liver Injury and Oxidative Stress

**Figure 2:**
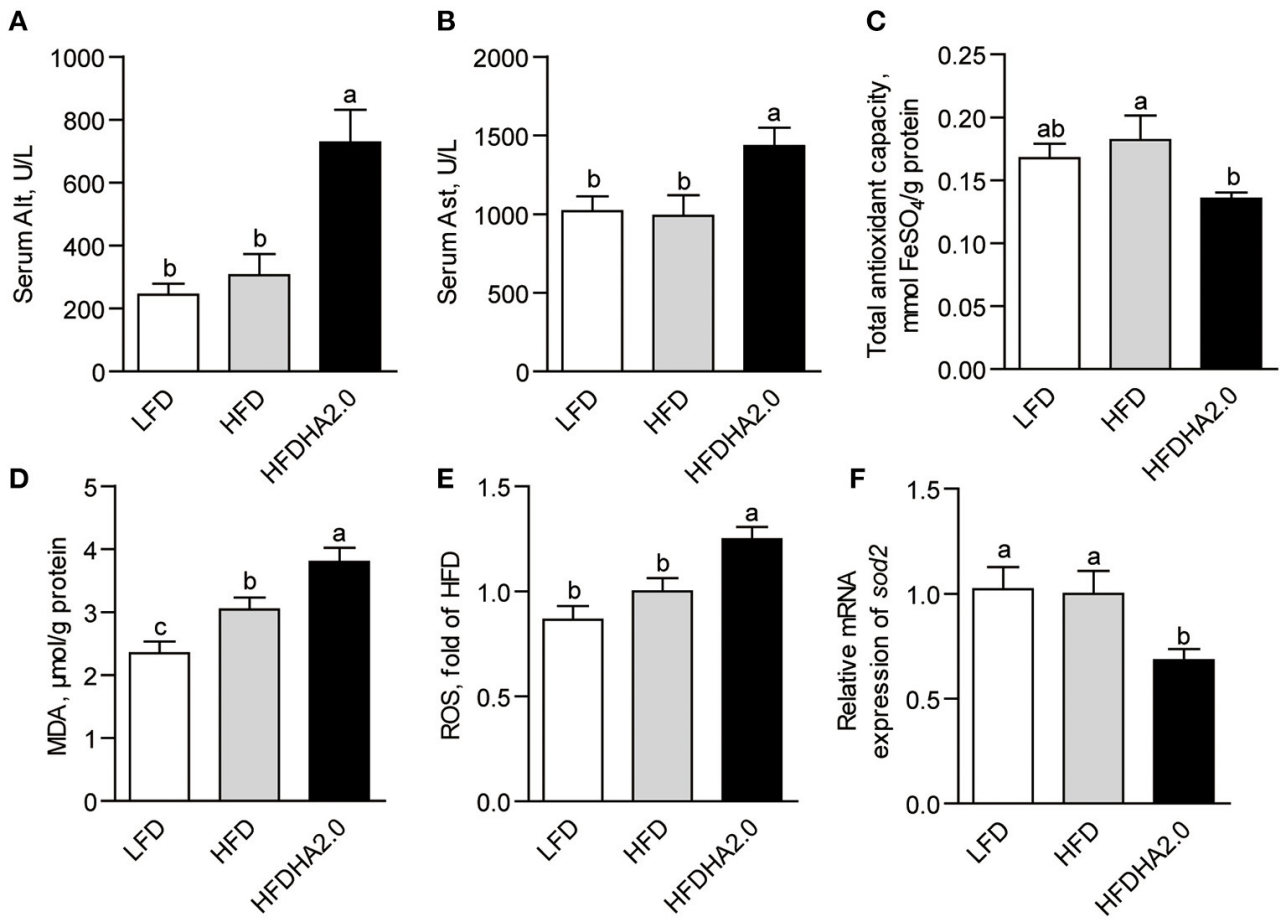
The effects of DHA on liver injury and oxidative stress in zebrafish. The activities of serum **(A)** Alt and **(B)** Ast of LFD, HFD or HFDHA2.0-fed zebrafish. Hepatic **(C)** T-AOC, **(D)** MDA and **(E)** ROS of LFD, HFD or HFDHA2.0-fed zebrafish. The relative mRNA expression of **(F)** *sod2* of LFD, HFD, or HFDHA2.0-fed zebrafish. The values are means ± SEMs (*n* = 5 or 6 biological replicates). The mean values without a common letter are significantly different, *p* < 0.05. LFD, low-fat diet; HFD, high-fat diet; HFDHA2.0, 2% DHA-supplemented HFD.

Zebrafish fed with HFDHA2.0 showed elevated serum Alt and Ast activities (2.4- and 1.4 -fold, respectively, *p* < 0.05; Figures 2A,B) as compared to that fed with HFD, suggesting 2% DHA supplementation induced liver injury. On the other hand, low DHA (0.5%)-supplemented HFD showed no significant effects on serum Alt and Ast activities (Supplementary Figures 3A,B). Meanwhile, LFDHA2.0 led to non-significant higher serum Alt and Ast activities when compared with LFD (Supplementary Figures 4A,B). Besides, zebrafish fed LFDHA2.0 showed a significant increase of T-AOC (*p* < 0.01) and unchanged MDA and ROS in the liver as compared to that fed with LFD (Supplementary Figures 4C,E). However, zebrafish fed with HFDHA2.0 had significantly reduced T-AOC and elevated MDA levels in the liver as compared to that fed with HFD (0.66- and 1.25-fold, respectively, *p* < 0.05; Figures 2C,D). Hepatic ROS was increased in the HFDHA2.0 group compared to that in the HFD group (*p* < 0.05; Figure 2E), while the mRNA expression of *sod2* was significantly inhibited by HFDHA2.0 feeding (0.68-fold, *p* < 0.05; Figure 2F). These results suggest that a high level of DHA could induce liver injury and hepatic oxidative stress when it is used in HFD. Thus, the following studies had focused on the negative effects of high dietary DHA in a high-fat formulation.

### The Effects of DHA on Apoptosis and Inflammation in the Liver

**Figure 3:**
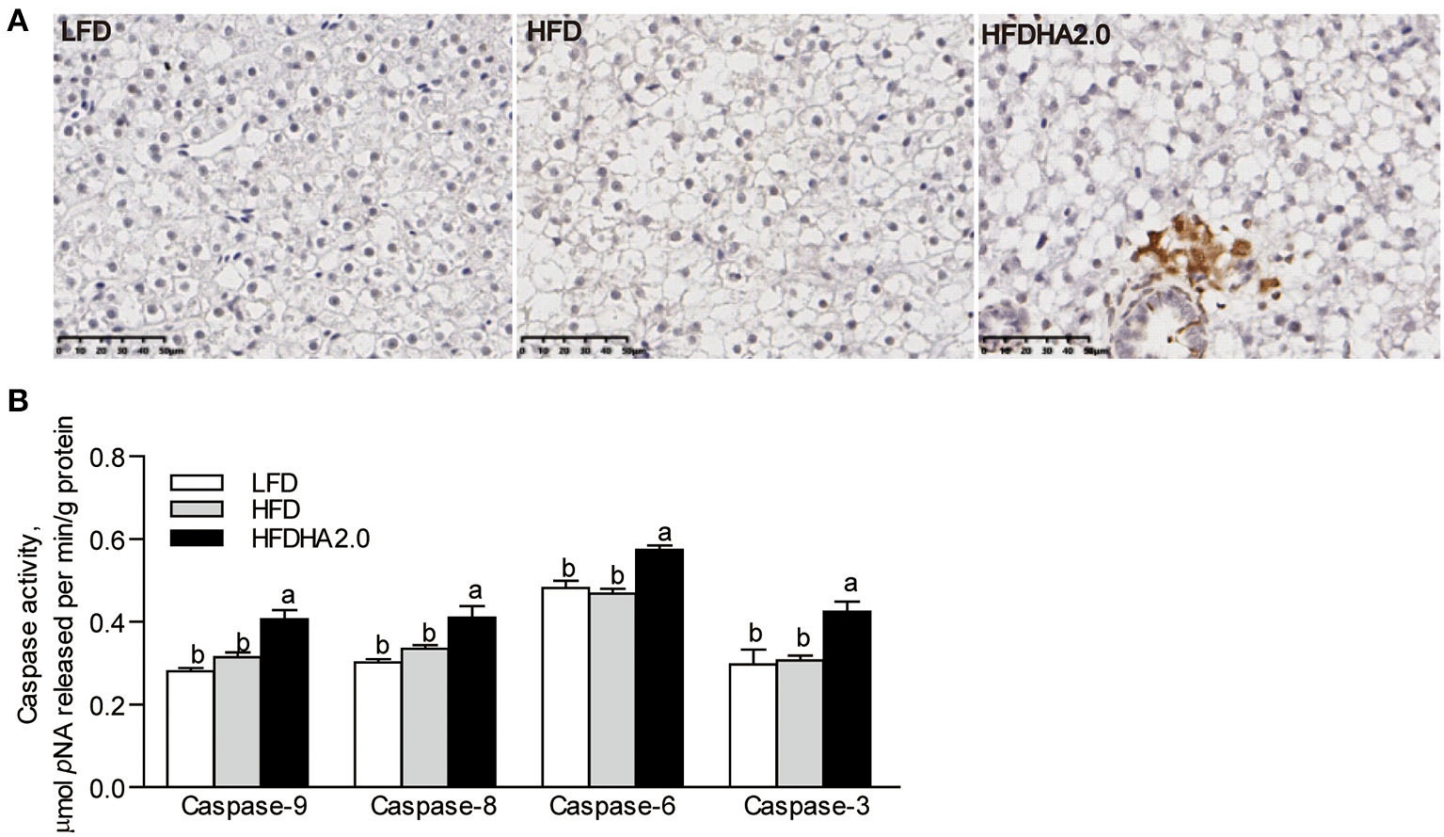
The effects of DHA on liver apoptosis in zebrafish. **(A)** Tunnel staining of liver sections of LFD, HFD, or HFDHA2.0-fed zebrafish. The scale bar is 50 μm. **(B)** The activities of caspase-9, caspase-8, caspase-6, and caspase-3 in LFD, HFD, or HFDHA2.0-fed zebrafish. The values are means ± SEMs (*n* = 6 biological replicates). The mean values without a common letter are significantly different, *p* < 0.05. LFD, low-fat diet; HFD, high-fat diet; HFDHA2.0, 2% DHA-supplemented HFD.

**Figure 4:**
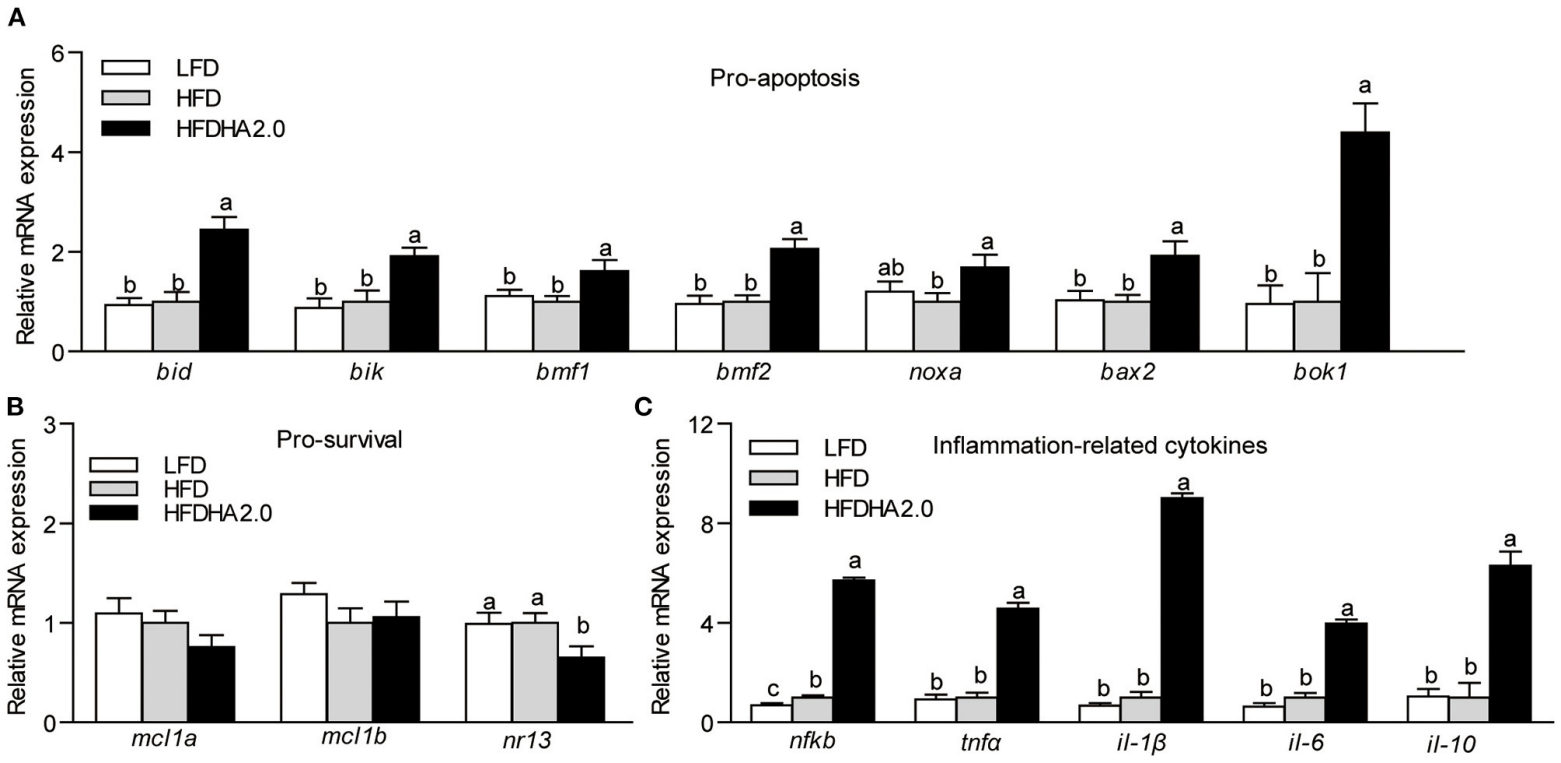
The effects of DHA on the relative mRNA expression of Bcl-2 family and inflammation-related cytokines in the livers of zebrafish. **(A)** The relative mRNA expression of pro-apoptotic proteins of LFD, HFD, or HFDHA2.0-fed zebrafish. **(B)** The relative mRNA expression of pro-survival proteins of LFD, HFD, or HFDHA2.0-fed zebrafish. **(C)** The relative mRNA expression of inflammatory cytokines of LFD, HFD, or HFDHA2.0-fed zebrafish. The values are means ± SEMs (*n* = 4–6 biological replicates). The mean values without a common letter are significantly different, *p* < 0.05. LFD, low-fat diet; HFD, high-fat diet; HFDHA2.0, 2% DHA-supplemented HFD.

According to tunnel staining of liver sections, fish fed with the HFDHA2.0 increased cell apoptosis compared with fish fed with HFD (Figure 3A). Furthermore, feeding the HFDHA2.0 for 2 weeks led to a moderate increase of capase-9 and caspase-3 activities as compared with HFD (Supplementary Figure 5A). Feeding HFDHA2.0 for 4 weeks led to a significant elevation of caspase-9, caspase-8, caspase-6, and caspase-3 activities when compared to the HFD (1.29-, 1.23-, 1.23- and 1.38-fold, respectively, *p* < 0.05; Figure 3B). Moreover, feeding the HFDHA2.0 upregulated the mRNA expression of pro-apoptotic genes *bid, bik, bmf1, bmf2, noxa, bax2*, and *bok1* (2.44-, 1.92-, 1.62-, 2.06-, 1.69-, 1.92- and 4.39-fold, respectively, *P* < 0.05; Figure 4A), while the mRNA expression of pro-survival *nr13* was downregulated (0.65-fold, *p* < 0.05; Figure 4B). All pro-inflammatory genes (*nfkb, tnf*α, *il-1*β, and *il-6*) were massively elevated in the livers of zebrafish fed with the HFDHA2.0 (5.70-, 4.57, 9.03, and 3.97-fold, respectively, *p* < 0.05; Figure 4C). This was also the case for the anti-inflammatory gene (*il-10*) (6.29-fold, *p* < 0.05; Figure 4C). These results suggest that a high level of DHA can induce cell apoptosis and inflammation.

### The Effects of DHA on Gut Microbiota Composition

**Figure 5:**
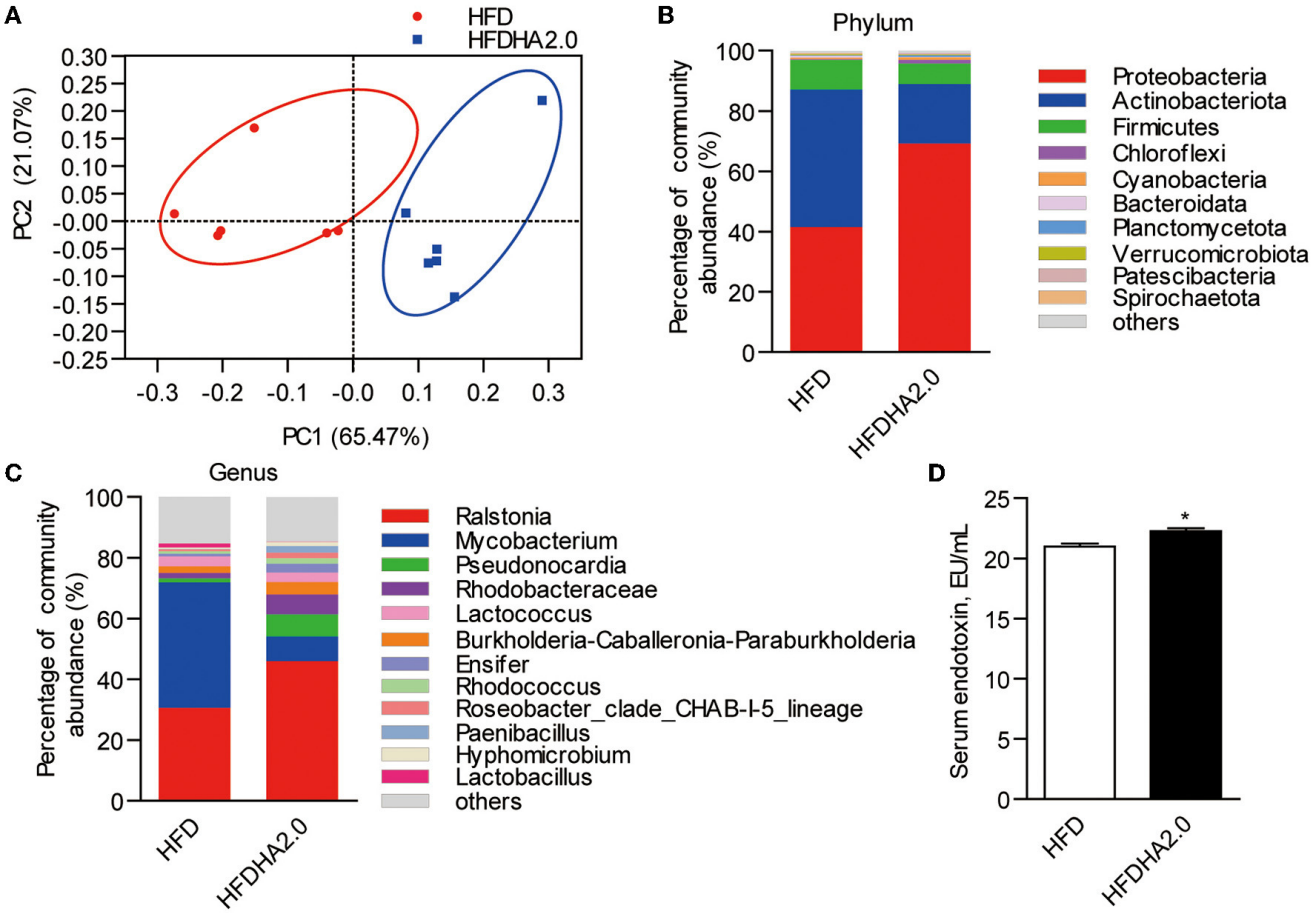
The effects of DHA supplementation on gut microbial community in zebrafish. **(A)** PCoA analysis of gut microbiota in HFD- or HFDHA2.0-fed zebrafish. **(B)** Relative abundance at the phylum level of gut microbial community from HFD- or HFDHA2.0-fed zebrafish. **(C)** Relative abundance at the genus level of gut microbial community from HFD- or HFDHA2.0-fed zebrafish. **(D)** Serum endotoxin in HFD- or HFDHA2.0-fed zebrafish. The values are means ± SEMs (*n* = 4 or 6 biological replicates). *p < 0.05. HFD, high-fat diet; HFDHA2.0, 2% DHA-supplemented HFD.

There were no significant differences in the intestinal microbiota α-diversity indexes between the HFD- and the HFDHA2.0-fed zebrafish (Table 4). By using Bray–Curtis distance, principal coordinate analysis (PCoA) analysis indicated the different gut community composition in OTU-level between the two groups with variances of PC1 65.47% and PC2 21.07% (Figure 5A). At the phylum level, a significant increase of the relative abundance of Proteobacteria (69.24 vs. 41.47%) and a decrease of relative abundance of Actinobacteria (19.71 vs. 45.66%) were observed in HFDHA2.0-fed zebrafish when compared with HFD-fed zebrafish (*p* < 0.01; Table 5; Figure 5B). At the genus level, *Raistonia* (45.91 vs. 30.58%) was significantly increased in the HFDHA2.0 group (*P* < 0.05), in contrast to *Mycobacterium* (8.15 vs. 41.31%) which was significantly decreased as compared with the HFD group (*P* < 0.01) (Table 6; Figure 5C). Accordingly, significantly higher serum endotoxin was observed in zebrafish fed with HFDHA2.0 compared to fish fed with HFD (*p* < 0.05; Figure 5D).

**Table 4:** Diversity index of gut bacteria of zebrafish fed with HFD or HFDHA2.0 for 4 weeks.

**Estimators**	**HFD**	**HFDHA2.0**
Shannon	2.29 ± 0.25	2.49 ± 0.16
Simpson	0.27 ± 0.03	0.26 ± 0.04
Ace	496.78 ± 77.01	439.28 ± 31.57
Chao	495.89 ± 79.03	440.99 ± 32.11

*Values are expressed as the mean ± SEM, n = 6. HFD, high-fat diet; HFDHA2.0, 2% DHA-supplemented HFD*.

**Table 5:** The predominant gut bacterial phylum in zebrafish fed with HFD or HFDHA2.0 for 4 weeks based on V3–V4 sequences.

**Phylum (%)**	**HFD**	**HFDHA2.0**
Proteobacteria	41.47 ± 4.24	69.24 ± 4.75^**^
Actinobacteriota	45.66 ± 3.05	19.71 ± 5.07^**^
Firmicutes	9.82 ± 5.07	6.76 ± 3.08
Chloroflexi	0.19 ± 0.05	1.20 ± 0.56
Cyanobacteria	0.47 ± 0.28	0.84 ± 0.24
Bacteroidata	0.69 ± 0.47	0.18 ± 0.04
Planctomycetota	0.15 ± 0.04	0.57 ± 0.33
Verrucomicrobiota	0.41 ± 0.23	0.27 ± 0.12

*The values are expressed as the mean ± SEM, n = 6.*

** *p < 0.01. HFD, high-fat diet; HFDHA2.0, 2% DHA-supplemented HFD*.

**Table 6:** The predominant gut bacterial genus in zebrafish fed with HFD or HFDHA2.0 for 4 weeks based on V3–V4 sequences.

**Genus (%)**	**HFD**	**HFDHA2.0**
*Ralstonia*	30.58 ± 3.65	45.92 ± 5.12^*^
*Mycobacterium*	41.31 ± 2.56	8.15 ± 4.75^**^
*Pseudonocardia*	1.33 ± 0.48	7.24 ± 3.56
*Rhodobacteraceae*	1.74 ± 0.58	6.51 ± 1.81^*^
*Burkholderia-Caballeronia-Paraburkholderia*	2.25 ± 0.37	4.14 ± 0.44^**^
*Lactococcus*	3.22 ± 1.06	3.14 ± 1.34
*Enfiser*	0.89 ± 0.26	2.89 ± 1.03
*Rhodococcus*	0.87 ± 0.12	1.83 ± 0.49
*Roseobacter_clade_CHAB-I-5_lineage*	0.60 ± 0.14	1.84 ± 0.63
*Paenibacillus*	0.04 ± 0.01	2.13 ± 2.26
*Hyphomicrobium*	0.54 ± 0.15	1.37 ± 0.46
*Lactobacillus*	1.36 ± 1.45	0.09 ± 0.04

*Values are expressed as the mean ± SEM, n = 6.*

* *p < 0.05,*

** *p < 0.01. HFD, high-fat diet; HFDHA2.0, 2% DHA-supplemented HFD*.

### The ZFL Cell Model: The Effects of High Level of DHA on Cell Viability and Apoptosis

**Figure 6:**
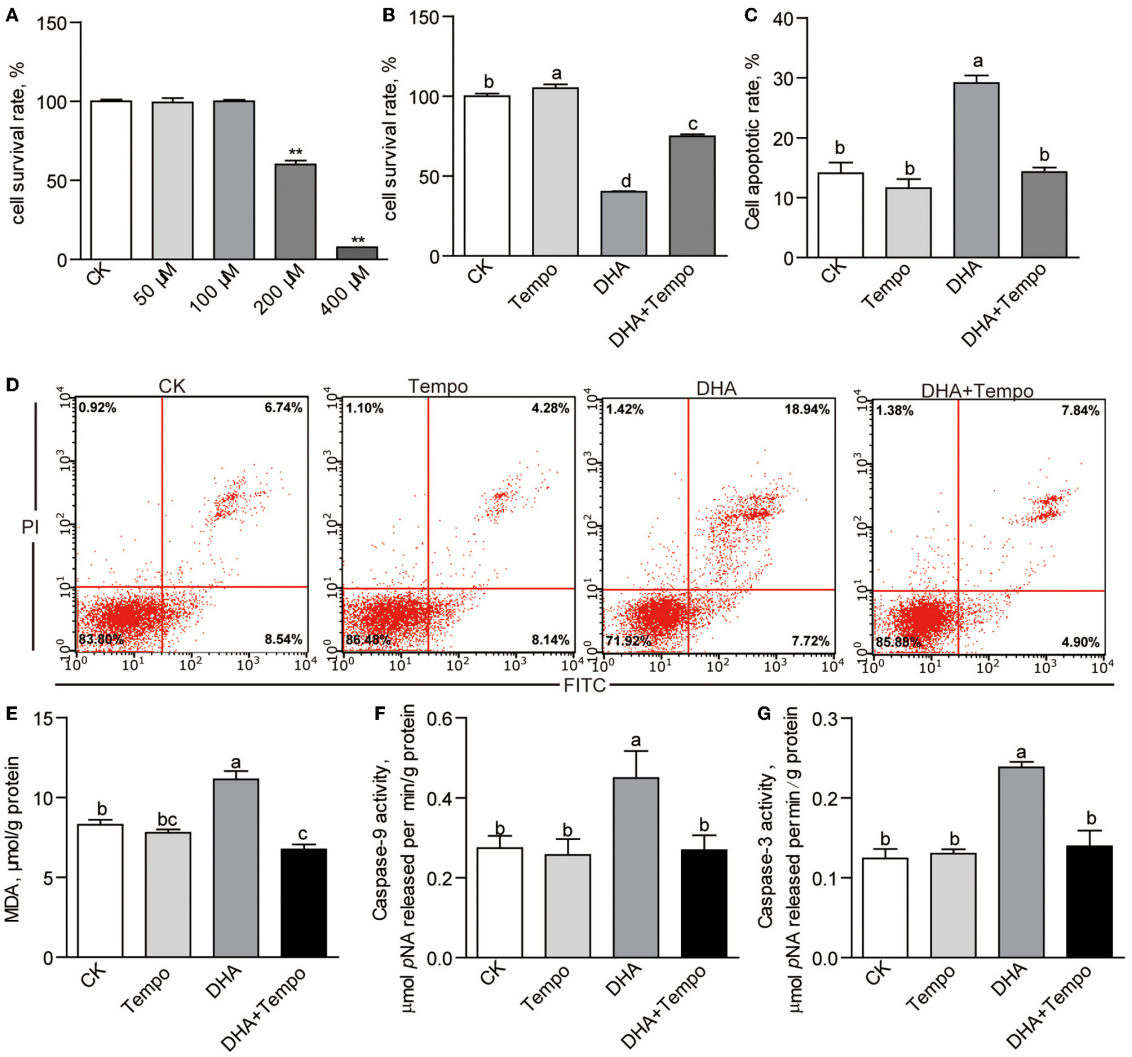
The effects of antioxidant, 4-hydroxy-Tempo (Tempo), on DHA cytotoxicity in ZFL cells. **(A)** The cell survival rates of ZFL cells treated with increasing concentrations of DHA for 24 h. **(B)** The cell survival rates and **(C)** the cell apoptotic rates of ZFL cells co-treated with DHA and tempo for 24 h. **(D)** The representative images of cell apoptosis of ZFL cells co-treated with DHA and tempo for 24 h. **(E)** The MDA levels in ZFL cells co-treated with DHA and tempo for 24 h. The activities of **(F)** caspase-9 and **(G)** caspase-3 in ZFL cells co-treated with DHA and tempo for 24 h. Values are means ± SEMs (*n* = 3 ~ 8 biological replicates). The mean values without a common letter are significantly different, *p* < 0.05. **p < 0.01.

The effects of DHA were then explored in a dose-response trial using ZFL cells with DHA concentrations from 0 to 400 μM. The viability test showed that DHA was nontoxic at concentrations up to 100 μM (Figure 6A). However, DHA reduced the cell survival rates to 60.07 and 7.66% at 200 and 400 μM, respectively (*p* < 0.01; Figure 6A). Thus, we adopted 200-μM DHA as the baseline of mildly challenged cells for further studies. Then control and 200-μM DHA-treated ZFL cells were co-treated with the antioxidant tempo. Tempo significantly improved the cell survival rate of 200-μM DHA-treated cells (*p* < 0.05; Figure 6B). Meanwhile, tempo reduced the cell apoptotic rate induced by 200-μM DHA (*p* < 0.05; Figures 6C,D). Besides, tempo reduced the elevated intracellular MDA level found in 200-μM DHA-treated cells (*p* < 0.05; Figure 6E). The activities of caspase-9 (*p* < 0.05; Figure 6F) and caspase-3 (*p* < 0.05; Figure 6G) were elevated by 200-μM DHA and reversed by addition of tempo. Caspase-8 activity was unaffected by 200-μM DHA or tempo (Supplementary Figure 5B).

### The ZFL Cell Model: The Effects of LPS on Cell Viability and Apoptosis

**Figure 7:**
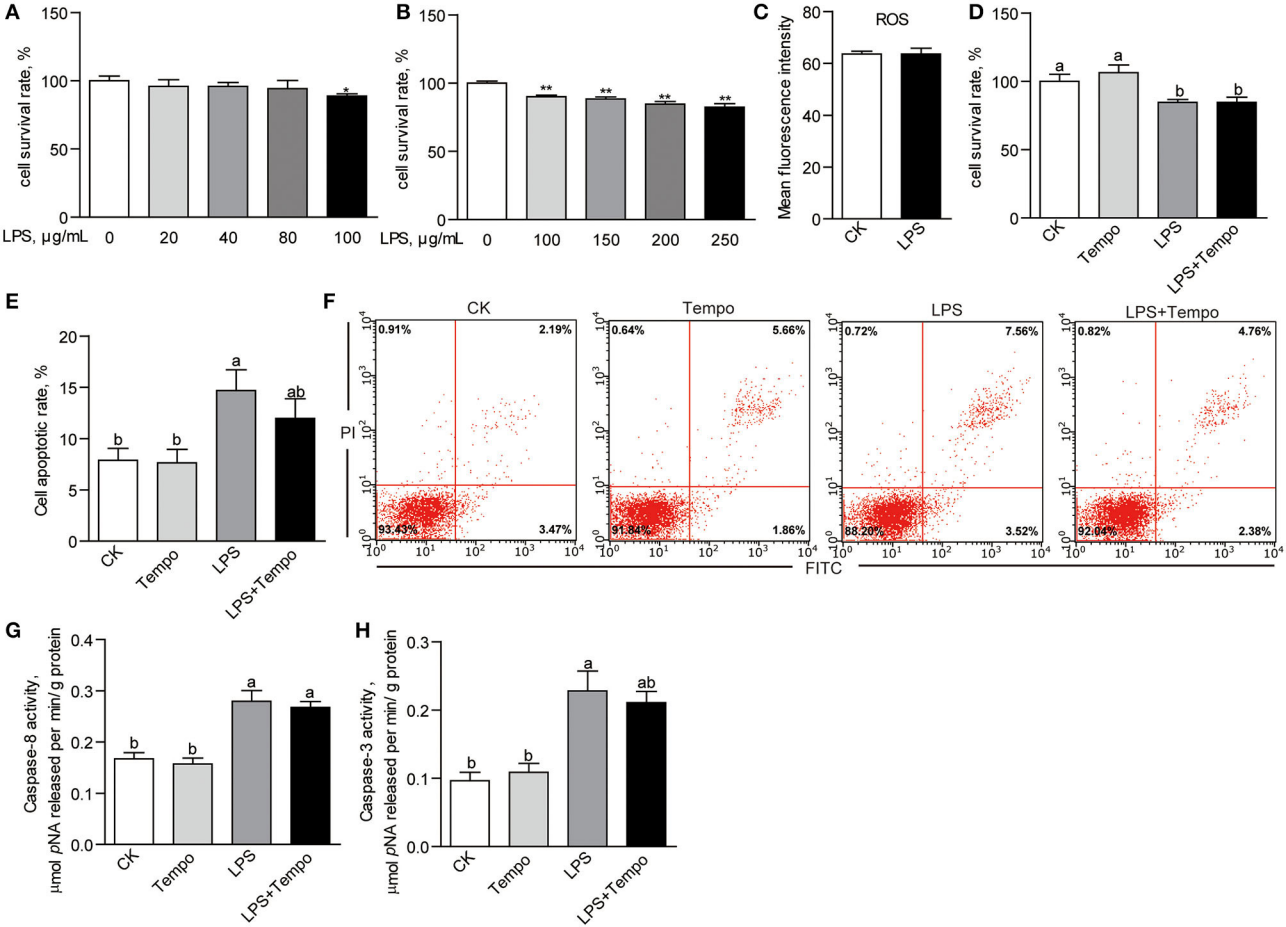
The effects of LPS on cell viability and apoptosis of ZFL cells. **(A)** The cell survival rates of ZFL cells treated with lower concentrations of LPS for 24 h. **(B)** The cell survival rates of ZFL cell treated with higher concentrations of LPS for 24 h. **(C)** The intracellular ROS of ZFL cells treated with 100 μg/ml LPS for 24 h. **(D)** The cell survival rates and **(E)** cell apoptotic rates of ZFL cells co-treated with LPS and tempo for 24 h. **(F)** The representative images of cell apoptosis of ZFL cells co-treated with LPS and tempo for 24 h. The activities of **(G)** caspase-8 and **(H)** caspase-3 in ZFL cells co-treated with DHA and tempo for 24 h. The values are means ± SEMs (*n* = 3–8 biological replicates). The mean values without a common letter are significantly different, *p* < 0.05. *p < 0.05, **p < 0.01.

Increasing the concentrations of LPS in culture media from 0 to 250 μg/ml gave a dual response in ZFL cells. At lower concentrations (20, 40, and 80 μg/ml) of LPS (*E. coli* O55: B5), there were no effects on cell viability compared to controls (Figure 7A). At higher concentrations (100, 150, 200, and 250 μg/ml), the suppression was dose-dependent (*p* < 0.01; Figures 7A,B). The 100-μg/ml LPS was used in the following experiments. However, results showed that 100-μg/ml LPS did not influence the production of intracellular ROS in ZFL cells (Figure 7C). Accordingly, tempo had no effect on the LPS-induced suppression of cell viability (Figure 7D). The LPS, on the other hand, induced a significant increase in cell apoptotic rate in ZFL cells (*p* < 0.05; Figures 7E,F). This was not affected by tempo (Figures 7E,F). Among the three initial caspases, LPS had no effect on the activity of caspase-9 (Supplementary Figure 5C), but significantly increased the activities of caspase-8 (*p* < 0.05; Figure 7G) and caspase-3 (*p* < 0.05; Figure 7H). Tempo had no effects on the activities of caspase-8 and caspase-3 in any of the groups.

## Discussion

DHA is particularly vulnerable to lipid peroxidation due to its five methylene-interrupted carbon atoms (39). This was clearly observed in the HFDHA2.0 group where both hepatic MDA and ROS levels increased when compared to the HFD group. Increased lipid peroxidation disturbs membrane organization and results in decreased fluidity (40–42), eventually leading to the activation of apoptosis (43–45). Mammalian studies suggest that the pro-apoptotic effects of DHA are involved with death receptor and mitochondria-dependent pathways (46–48). In addition, DHA hydroperoxides can induce apoptosis through a secondary mitochondrial dysfunction pathway which is associated with lipid peroxidation reaction (49, 50). The DHA peroxidation product, 4-hydroxyhexenal, is a potent inducer of mitochondrial permeability (51) that has been suggested as a central coordinating event of apoptosis (52). These evidence can support the increased tunnel-positive cells and caspase-9, caspase-8, caspase-6, and caspase-3 activities in the HFDHA2.0 group.

Caspase-9 is the initiator caspase of the intrinsic apoptosis pathway (53). The members of the Bcl-2 protein family control this pathway (54). The activation of caspase-9 and its effector caspases depends on the release of pro-apoptotic proteins from the mitochondria upon apoptotic stimulation (54, 55). The observed upregulated expression of pro-apoptotic genes in the HFDHA2.0 group further demonstrates excess DHA can activate the mitochondrial death pathway in zebrafish liver. Caspase-8 is the initiator caspase of death receptor-induced apoptosis (56), moreover, it is required for inflammatory signaling (57–59). Thus, the increased caspase-8 activity and up-regulation of *nfkb* and inflammatory cytokines in the HFDHA2.0 group suggest that excess dietary DHA could activate death receptor-induced apoptosis in the liver. However, when validated in the ZFL cell model, DHA only stimulated the elevation of caspase-9 activity, which can be inhibited by the antioxidant, tempo. This suggests that DHA itself activates the mitochondrial death pathway through lipid peroxidation, while other factors might contribute to the activation of caspase-8-mediated death receptor-induced apoptosis *in vivo*.

The effects of dietary lipids on gut microbiota depend on their types and levels in diets (60–62). Previous studies have suggested that HFDs tend to disrupt gut microbiota composition (63, 64) whereas dietary *n*-3 LC-PUFAs exert positive effects by reverting these microbiota dysbiosis (17, 65). However, it has also been suggested that high *n*-3 LC-PUFAs in high-lipid formulation can induce an unhealthy gut microbiota community characterized by the reduction of lactic acid bacteria and higher abundance of *Mycoplasma, Burkholderiaceae, Bacteroidales*, and *Ralstonia* in Atlantic Salmon (*S. salar*) and hybrid grouper (female *E. fuscoguttatus* × male *E. lanceolatu*) (26, 27). Similarly, high dietary DHA in a high-lipid formulation was shown to lead to the enrichment of Proteobacteria, *Ralstonia, Rhodobacteraceae*, and *Burkholderia–Caballeronia–Paraburkholderia* in this study.

Proteobacteria consists of gram-negative bacteria with LPS in the outer leaflet of its bilayer structure and it can constantly release LPS to its surroundings in a form of outer membrane vesicles (66, 67). Besides, gut microbiota-derived LPS can enter the liver *via* portal blood flow and lead to inflammation-based disorders (68, 69). Additionally, the members of Proteobacteria are facultative anaerobes, suggesting that oxygen concentration in the gut can influence their abundance (70). A healthy intestinal mucosa is characterized by a steep oxygen gradient along the length of the intestine and from the lumen to the serosa, namely, “physiologic hypoxia” (71). Therefore, the increase of Proteobacteria in the HFDHA2.0 group likely reflects less healthy gut. Importantly, the alteration of gut microbiome and increased intestinal permeability are the two reasons for elevated serum LPS (72). In this study, we observed significantly elevated serum endotoxin in zebrafish fed with HFDHA2.0. Thus, we speculated that the enrichment of Proteobacteria induced by a high level of DHA might mediate the activation of caspase-8 through the production of LPS. To validate the effects of LPS on liver health, we measured cell viability and apoptosis in ZFL cells treated with LPS. In this study, LPS-treated cells had elevated apoptotic rate and caspase-8 activity, likely reflecting the activation of death receptor apoptosis and consistent with the results observed *in vivo*. Moreover, antioxidant tempo cannot inhibit the elevation of caspase-8 activity, showing that the pro-apoptotic effects of LPS are independent of lipid peroxidation. Interestingly, the inflammation signal activated by LPS promotes the *de novo* lipogenesis and it inhibits fatty acid β-oxidation, leading to hepatic lipid accumulation (73, 74). The elevated serum endotoxin might partly account for the ineffectiveness of a high level of DHA on hepatic lipid accumulation.

The cytotoxicity of DHA is controversial. On one hand, DHA can protect myotubes and colonic epithelial cells from atrophy induced by palmitate and arabinose operon regulatory protein (araC), respectively (75, 76). The DHA ameliorates hepatocyte apoptosis induced by oleic acid in grass carp (77). On the other hand, DHA induces cell apoptosis in many mammalian cell types, including adipocytes, colonocytes, cardiac cells, and vascular smooth muscle cells (14, 78–80). Even though DHA plays important roles in the structure and functions of cell membranes (81), its highly unsaturated property makes it susceptible to peroxidation, further leading to free radical chain reactions (82). Free radicals are molecules with one or more unpaired electrons and exist in the form of ROS (83). At the physiological level, ROS serves as a signal transducer in a myriad of intracellular signaling pathways (84), whereas excess ROS induces cellular oxidative damage (84). Although the reasons for the paradoxical results in DHA have not been clarified, the “baseline of ROS” in different cell types or the same cell type cultured in different systems might account for the susceptibility to DHA cytotoxicity. For example, many studies have suggested that DHA is a potential anti-cancer nutrient, with its dramatic damage to various of cancer cells, including human hepatocellular carcinoma cells, colon cancer cells, and breast cancer cells (46, 85, 86). During tumorigenesis, the ability of cancer cells to generate ROS is greatly increased (87, 88), which means the probability of DHA being attacked by free radicals is higher than that in normal tissues. Thus, DHA can present remarkable effects on tumor control and anti-cancer (89, 90). In this study, DHA was used in a high-lipid formulation. Although HFD feeding in the present study did not induce a significant change in the activities of serum enzymes related to liver injury and hepatic enzymes related to apoptosis, increasing levels of MDA and ROS suggest the potential role of HFD in unbalancing hepatic redox status and sensitizing DHA to peroxidation. Furthermore, the long-term feeding trials (9~12 weeks) in rats have suggested that HFD can lead to significant increase in hepatic lipid peroxidation (91–93). This indicates that the HFD feeding in the current study might modify the sensibility of liver to DHA-initiated lipid peroxidation.

In conclusion, a high level of dietary DHA can compromise the liver health of zebrafish in the context of HFD. This study indicates that excess DHA supplementation of HFD has an aggravated effect on hepatocellular apoptosis-related injury, which might go through at least two different modes, including DHA-initiated lipid peroxidation and gut microbiota-generated LPS. First, DHA molecular activates the intrinsic apoptosis pathway through lipid oxidation. Second, excess dietary DHA promotes the growth of Proteobacteria that might account for the activation of death receptor-induced apoptosis and inflammation by releasing LPS. The HFD formulation might increase the sensibility of the liver to DHA-initiated lipid peroxidation in zebrafish. This study also suggests that exogenous antioxidant is important for DHA supplementation in feeds.

## Data Availability Statement

The datasets presented in this study are deposited in the National Center for Biotechnology Information (NCBI), accession number PRJNA803766.

## Ethics Statement

The animal study was reviewed and approved by Institute of Feed Research of Chinese Academy of Agricultural Sciences Animal Care Committee.

## Author Contributions

ZZho designed the research. QD wrote the article, performed experiments, and acquired data. ZZha gave conceptual advice for the article and assisted in gut microbiota analysis. ER and RO reviewed and helped to revise the manuscript. QH and QZ participated in zebrafish husbandry and sampling. CR, YY, and ZZha co-analyzed and discussed the results. All authors read and approved the final manuscript.

## Funding

This work was supported by the National Natural Science Foundation of China (NSFC 31925038 and 32061133004).

## Conflict of Interest

The authors declare that the research was conducted in the absence of any commercial or financial relationships that could be construed as a potential conflict of interest.

## Publisher's Note

All claims expressed in this article are solely those of the authors and do not necessarily represent those of their affiliated organizations, or those of the publisher, the editors and the reviewers. Any product that may be evaluated in this article, or claim that may be made by its manufacturer, is not guaranteed or endorsed by the publisher.
