# Supplementary material for: Excess DHA Induces Liver Injury via Lipid Peroxidation and Gut Microbiota-Derived Lipopolysaccharide in Zebrafish
Source: Front Nutr. 2022 Apr 28;9:870343. doi: 10.3389/fnut.2022.870343 (PMC9096794; doi:10.3389/fnut.2022.870343)
Supplement: Supplementary file 1 [file Data_Sheet_1.pdf]

Supplemental Figure

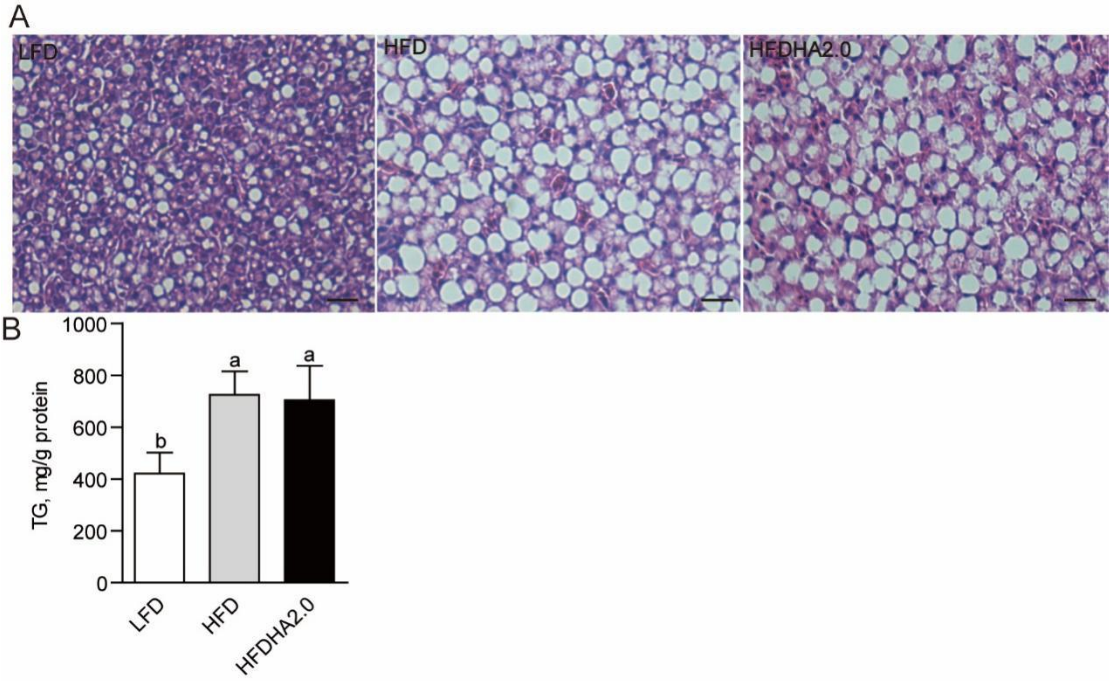

Supplemental Figure 1. (A) H&E staining of liver sections of LFD, HFD or HFDHA2.0-fed zebrafish. The scale bar for H&E staining is 20 μm. (B) Hepatic TG contents of zebrafish fed LFD, HFD or HFDHA2.0. Values are means ± SEMs (n=3 biological replicates). Means without a common letter are significantly different, $P < 0.05$. LFD, low fat diet; HFD, high-fat diet; HFDHA2.0, 2% DHA-supplemented HFD.

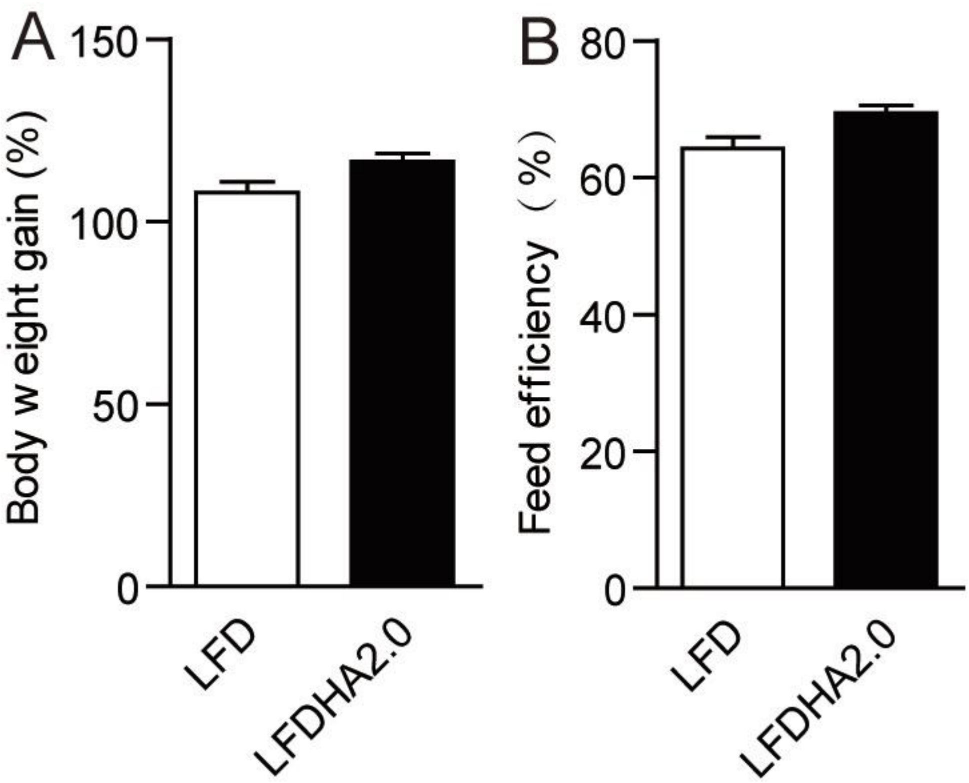

Supplemental Figure 2. (A) Body weight gain and (B) feed efficiency of LFD or LFDHA2.0-fed zebrafish. Values are means ± SEMs (n=3 biological replicates). LFD, low fat diet; LFDHA2.0, 2% DHA-supplemented LFD.

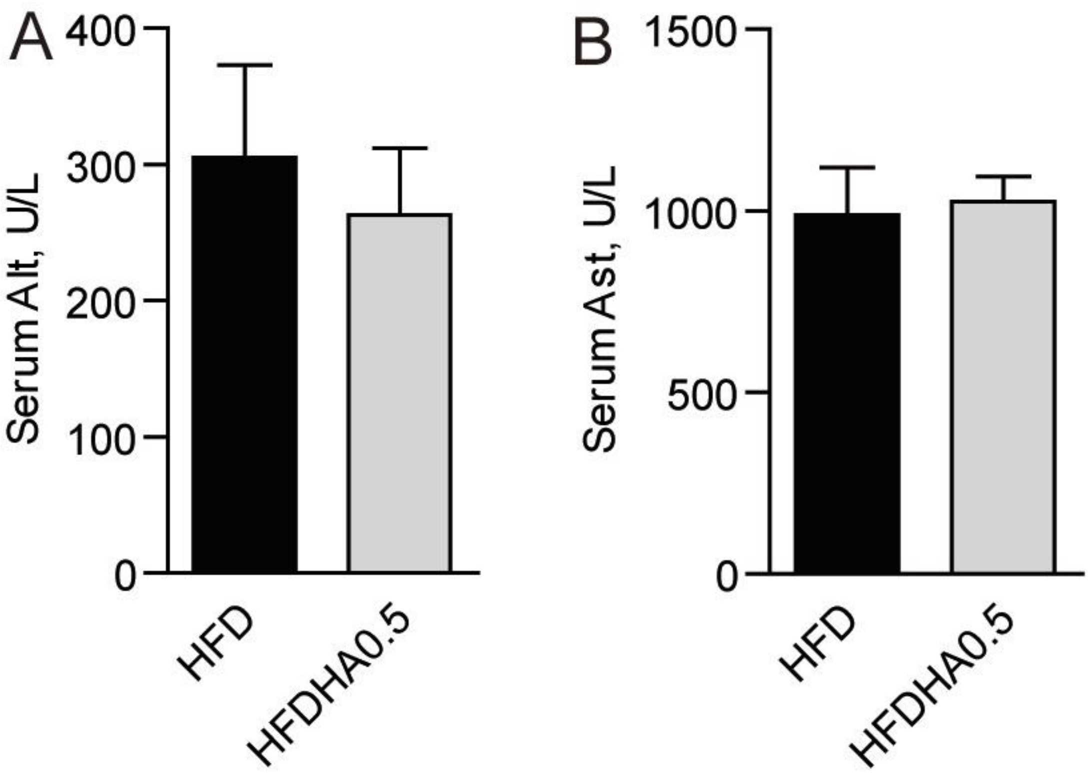

Supplemental Figure 3. The activities of serum (A) Alt and (B) Ast of HFD or HFDHA0.5-fed zebrafish. Values are means ± SEMs (n=5 biological replicates). HFD, high fat diet. HFDHA0.5, 0.5% DHA-supplemented high fat diet.

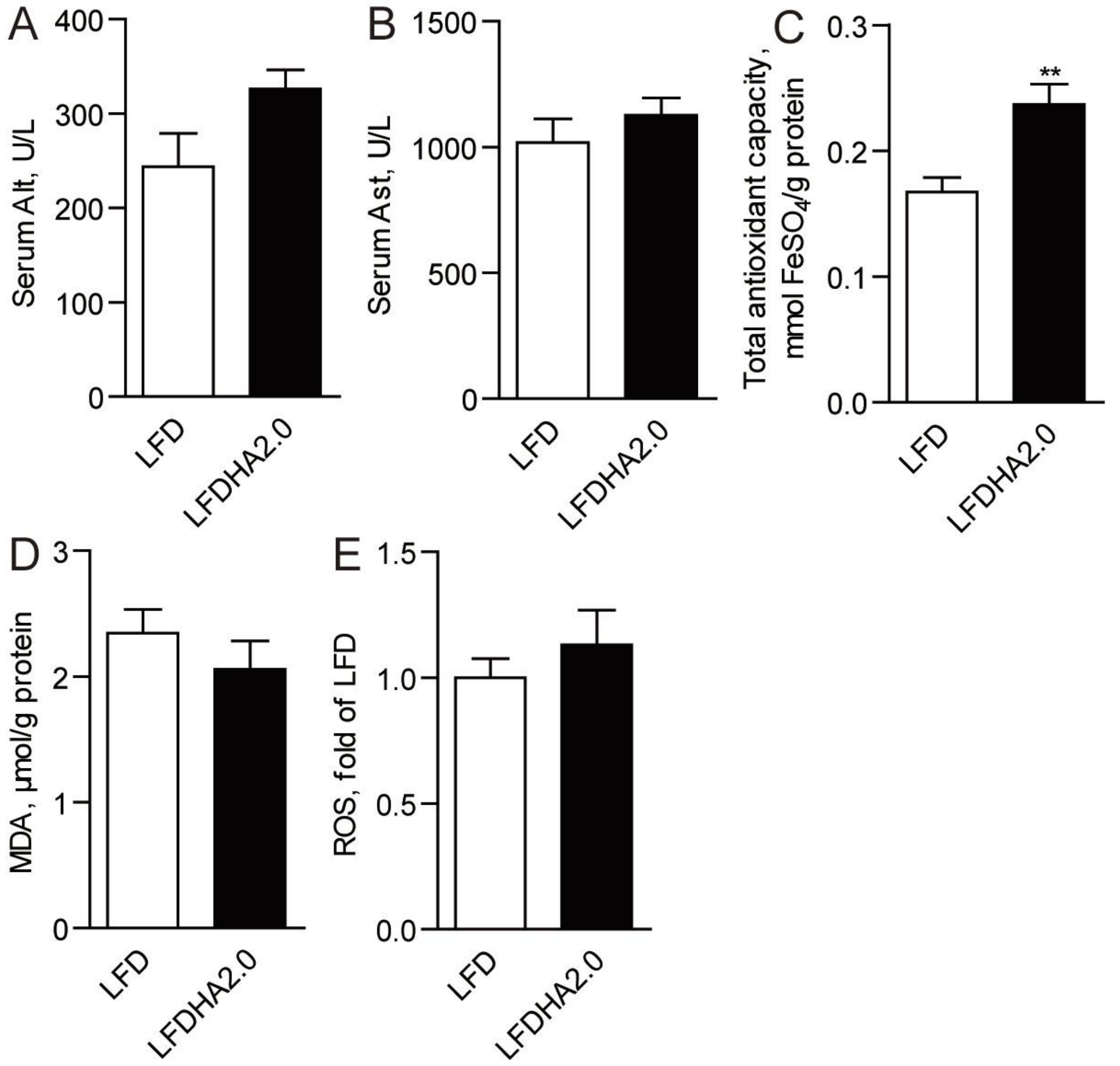

Supplemental Figure 4. The activities of serum (A) Alt and (B) Ast of LFD or LFDHA2.0-fed zebrafish. Hepatic (C) T-AOC, (D) MDA and (E) ROS of LFD or LFDHA2.0-fed zebrafish. Values are means ± SEMs (n=3~6 biological replicates). **, $P < 0.01$. LFD, low fat diet; LFDHA2.0, 2% DHA-supplemented LFD.

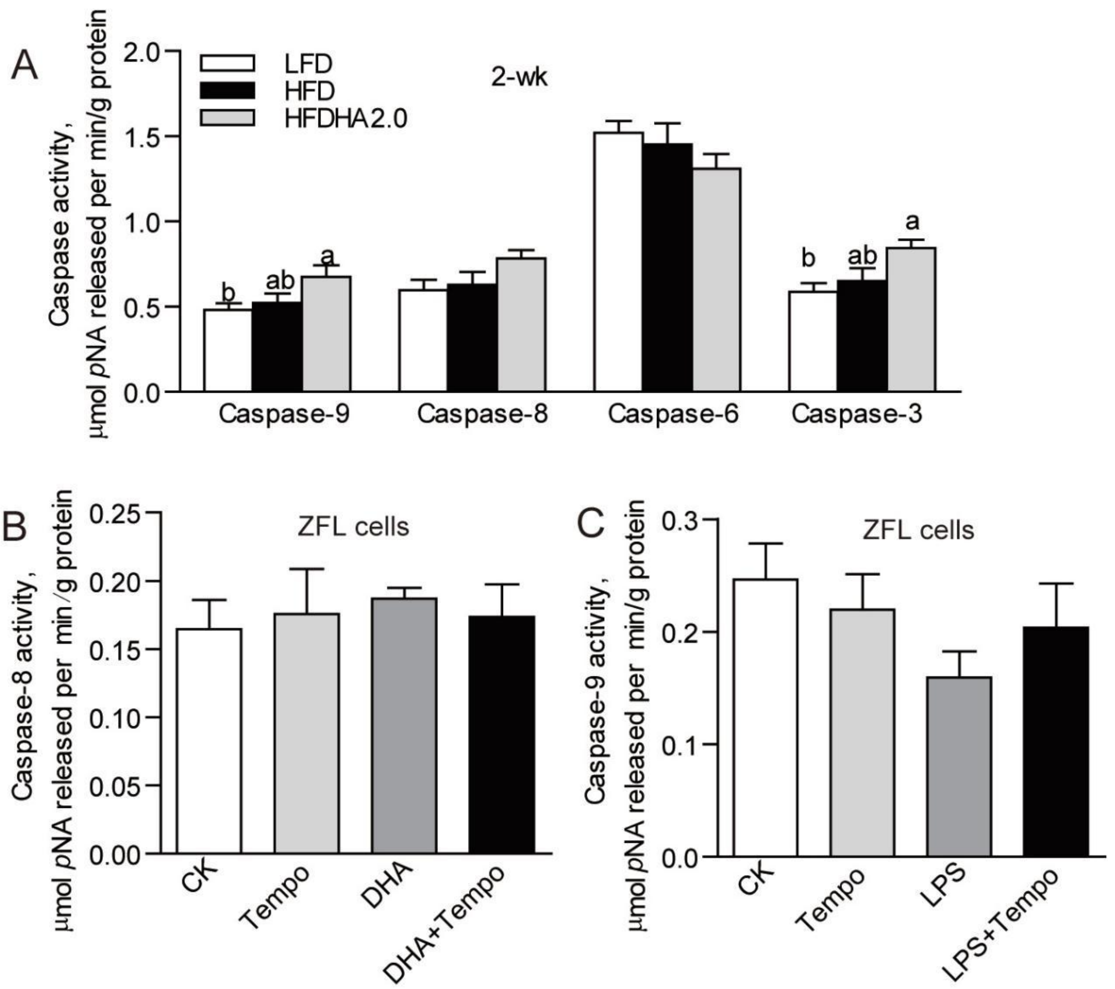

Supplemental Figure 5. (A) The activities of caspase-9/8/6/3 in the liver of LFD and HFD or HFDHA2.0-fed zebrafish at 2 weeks. (B) The activity of caspase-8 in ZFL cells co-treated with DHA and 4-hydroxy-tempo (tempo) for 24 h. (C) The activity of caspase-9 in ZFL cells co-treated with DHA and tempo for 24 h. Values are means ± SEMs (n=5 or 6 biological replicates). Means without a common letter are significantly different, $P < 0.05$. LFD, low fat diet; HFD, high fat diet; HFDHA2.0, 2% DHA-supplemented HFD. LPS, lipopolysaccharide.
